# Outcomes in clinical subgroups of patients with alcohol-related hospitalizations: a population-based retrospective cohort study

**DOI:** 10.23889/ijpds.v9i5.2699

**Published:** 2024-09-18

**Authors:** Erik Friesen, Andrea Mataruga, Nathan Nickel, Paul Kurdyak, James Bolton

**Affiliations:** 1 Temerty Faculty of Medicine, University of Toronto Centre for Addiction and Mental Health (CAMH); 2 Centre for Addiction and Mental Health (CAMH); 3 Mental Health and Addiction Research Program, ICES; 4 Manitoba Centre for Health Policy; 5 Department of Psychiatry, Max Rady College of Medicine, University of Manitoba

## Objective and Approach

Individuals who experience alcohol-related hospitalizations are at a high risk of recurrent harm and premature mortality. This project characterized the clinical subgroups of individuals who experience alcohol-related hospitalizations to understand who is at the highest risk of recurrent harm following discharge.

## Approach

Population-based retrospective cohort study of individuals with an alcohol-related hospitalization between 2017-2018 in two Canadian provinces (Ontario and Manitoba) using linked provincial health administrative databases. Clinical subgroups were identified with latent class analysis based on the type and frequency of alcohol-related health service use in the two-years preceding the index hospitalization. Associations between subgroup membership, readmission, and mortality in the year following discharge were evaluated using multivariable time-to-event regression.

## Results

In cohorts of 4,753 (Manitoba) and 29,290 (Ontario) individuals, seven subgroups were identified. These followed a severity gradient from low-frequency service use for acute intoxication to high-frequency service use for alcoholic liver disease. Individuals in the ‘liver disease’ subgroup had the highest risk of 1-year mortality relative to the rest of the cohort (adjusted hazard ratio [aHR]: 3.83, 95% confidence interval (CI): 2.80-5.24). A small subgroup of individuals with a history of high-frequency alcohol-related health service had the highest hazard of readmission (aHR: 5.09, 95% CI: 4.11-6.31).

## Conclusions and Implications

There are distinct clinical subgroups of individuals who experience alcohol related hospitalizations and individuals with high-frequency health service use and alcohol-related liver disease are at the highest risk of readmission and mortality. These subgroups merit consideration in strategies aimed at reducing the risk of post-discharge harm.

